# Clinical Significance of Annexin A2 Expression in Breast Cancer Patients

**DOI:** 10.3390/cancers13010002

**Published:** 2020-12-22

**Authors:** Lee D. Gibbs, Kelsey Mansheim, Sayantan Maji, Rajesh Nandy, Cheryl M. Lewis, Jamboor K. Vishwanatha, Pankaj Chaudhary

**Affiliations:** 1 Department of Microbiology, Immunology and Genetics, Graduate School of Biomedical Sciences, University of North Texas Health Science Center, Fort Worth, TX 76107, USA; leedanig@usc.edu (L.D.G.); sayantan.maji@live.unthsc.edu (S.M.); Jamboor.vishwanatha@unthsc.edu (J.K.V.); 2 Department of Pathology, Brookwood Baptist Health, 1130 22nd St S # 1000, Birmingham, AL 35205, USA; kelsey.mansheim@bswhealth.org; 3 Department of Biostatistics and Epidemiology, School of Public Health, University of North Texas Health Science Center, Fort Worth, TX 76107, USA; Rajesh.Nandy@unthsc.edu; 4 Simmons Comprehensive Cancer Center, University of Texas Southwestern Medical Center, Dallas, TX 75390, USA; cheryl.lewis@utsouthwestern.edu; 5 Center for Diversity and International Programs, University of North Texas Health Science Center, Fort Worth, TX 76107, USA

**Keywords:** breast cancer, annexin A2, biomarker, TNBC

## Abstract

**Simple Summary:**

Annexin A2 (AnxA2) is a Ca^++^-dependent phospholipid-binding protein that is involved in invasion and metastasis of breast cancer. However, the expression of AnxA2 in breast cancer patients has not been reported. Here, we show that the expression of AnxA2 was high in tumor tissues and serum samples of breast cancer patients compared to non-cancer patients. The high expression of serum AnxA2 in breast cancer was associated with tumor grade and poor survival. The expression and diagnostic value of serum AnxA2 was high in triple-negative breast cancer (TNBC) subtypes and associated with the phosphorylation of AnxA2 at tyrosine 23. Overall, this study highlights the diagnostic and prognostic significance of AnxA2 in breast cancer.

**Abstract:**

Increasing evidence suggests that AnxA2 contributes to invasion and metastasis of breast cancer. However, the clinical significance of AnxA2 expression in breast cancer has not been reported. The expression of AnxA2 in cell lines, tumor tissues, and serum samples of breast cancer patients were analyzed by immunoblotting, immunohistochemistry, and enzyme-linked immunosorbent assay, respectively. We found that AnxA2 was significantly upregulated in tumor tissues and serum samples of breast cancer patients compared with normal controls. The high expression of serum AnxA2 was significantly associated with tumor grades and poor survival of the breast cancer patients. Based on molecular subtypes, AnxA2 expression was significantly elevated in tumor tissues and serum samples of triple-negative breast cancer (TNBC) patients compared with other breast cancer subtypes. Our analyses on breast cancer cell lines demonstrated that secretion of AnxA2 is associated with its tyrosine 23 (Tyr23) phosphorylation in cells. The expression of non-phosphomimetic mutant of AnxA2 in HCC1395 cells inhibits its secretion from cells compared to wild-type AnxA2, which further suggest that Tyr23 phosphorylation is a critical step for AnxA2 secretion from TNBC cells. Our analysis of AnxA2 phosphorylation in clinical samples further confirmed that the phosphorylation of AnxA2 at Tyr23 was high in tumor tissues of TNBC patients compared to matched adjacent non-tumorigenic breast tissues. Furthermore, we observed that the diagnostic value of serum AnxA2 was significantly high in TNBC compared with other breast cancer subtypes. These findings suggest that serum AnxA2 concentration could be a potential diagnostic biomarker for TNBC patients.

## 1. Introduction

Breast cancer is the most common and second leading cause of cancer-related deaths among North American women, with the expectation that 276,480 new cases will be diagnosed with 42,170 deaths in 2020 [1]. Breast cancer is a diverse and heterogeneous disease with different phenotypes, prognosis, and responses to treatment [2,3,4,5,6]. Breast cancer is primarily characterized by the expression of estrogen receptor (ER), progesterone receptor (PR), and/or human epidermal growth factor receptor type 2 (HER2) [7,8]. The status of these three markers and other clinicopathological factors such as age, race, menopause status, tumor grade and stage, and lymph node involvement have been identified as prognostic factors that help in making decisions on systemic therapies [6,9]. Although several efforts have been made to improve the therapeutic strategies, the clinical outcome of breast cancer patients remains unsatisfactory due to diseases recurrence and metastasis. Thus, there is still an urgent need to establish novel noninvasive biomarkers which are easy to detect and valuable for prognostic and diagnostic prediction.

Annexin A2 (AnxA2) is a calcium-dependent anionic phospholipid binding protein and involved in mediating several intracellular actions of calcium [10,11]. The C-terminal domain of AnxA2 contains the anionic phospholipid- and Ca^++^-binding properties, whereas the N-terminus is the site for posttranslational modifications and interactions with other proteins [12,13]. The N-terminus possesses phosphorylation sites on tyrosine 23 (Tyr23), which is a substrate for phosphorylation by Src kinase [14,15] and serine 25 (Ser25), which has been reported to be phosphorylated by protein kinase C [16,17]. AnxA2 exists as a monomer in the cytosol and as a heterotetrameric complex with S100A10 [(AnxA2)2-(S100A10)2] at the cell surface [18,19,20]. Phosphorylation of AnxA2 at Tyr23 is an important event for the localization of AnxA2 to the extracellular cell surface and has a key role in several membrane-related events, including fibrinolysis, ion channel conductance, cell-cell adhesion, exocytosis and endocytosis, and membrane-cytoskeletal interactions [14,18,19,20,21,22,23]. At the cell surface, AnxA2 heterotetramer complex provides binding site for both plasminogen and tissue type plasminogen activator (tPA) and converts plasminogen into plasmin [24,25,26,27], which plays an important role in invasion and metastasis of cancer. AnxA2 is overexpressed in tumor tissues of both invasive ductal mammary carcinoma and ductal carcinoma in situ compared to normal and hyperplastic ductal epithelial cells and ductal complexes, suggesting a crucial role of AnxA2 in breast tumor malignancy and invasiveness [24,25,26,27,28]. AnxA2 is abundantly overexpressed in TNBC cell lines and has a reciprocal relationship with HER2 [29]. Moreover, we have previously shown that AnxA2 gene is overexpressed in tumor tissues of triple-negative breast cancer compared to ER^+^, PR^+^, and HER2^+^ breast cancer patients [30,31]. In addition, the overexpression of AnxA2 gene is positively correlated with poor outcomes of the patients with TNBC [30,31]. In the extracellular environment, AnxA2 secretion is correlated with the invasive phenotype of the breast cancer cells [32]. Although these previous studies revealed AnxA2 as an important player for breast cancer progression, the expression of AnxA2 protein in tumor tissues of different subtypes of breast cancer patients and its role as a secretory biomarker for breast cancer prognosis and diagnosis has not been studied.

In the present study, we analyzed the expression of AnxA2 in tumor tissues of different subtypes of breast cancer patients and normal breast tissues. In addition, to explore the diagnostic and prognostic value of serum AnxA2 levels in breast cancer patients, we examined the expression of serum AnxA2 levels in different subtypes of breast cancer patients and normal healthy females by enzyme-linked immunosorbent assay (ELISA), and evaluated the expression of serum AnxA2 levels with diagnosis and prognosis of breast cancer patients. Subsequently, we established that the high expression of AnxA2 seen in sera of TNBC patients is associated with the phosphorylation status of AnxA2 at Tyr23 in tumor cells. These data suggest that serum AnxA2 may serve as a diagnostic and prognostic serological biomarker for TNBC patients.

## 2. Results

### 2.1. AnxA2 Expression in Tumor Tissues of Breast Cancer Patients

**Figure 1:**
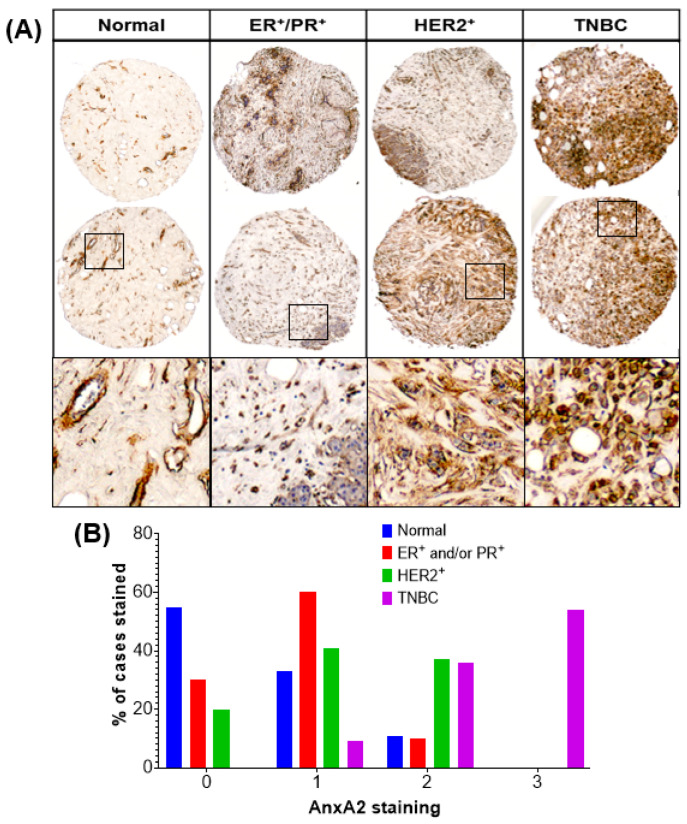
Immunohistochemical analysis of AnxA2 expression in different subtypes of breast cancer tissue and normal breast tissue specimens. (**A**) Paraffin embedded tissue sections were stained with AnxA2 monoclonal antibody. Representative images of normal, ER^+^/PR^+^, HER2^+^, and TNBC tumor tissue specimens showing status of AnxA2 expression. AnxA2 was primarily localized to the plasma membrane of tumor cells in TNBC specimens. (**B**) Bar chart showing the AnxA2 staining patter in normal and different subtypes of breast cancer tissue specimens (Chi square test: χ² = 50.54, *p* < 0.0001).

The expression of AnxA2 protein were analyzed in 67 breast tumor tissues and 9 normal breast tissues by immunohistochemistry (IHC) analysis. The intensity of the AnxA2 staining was categorized as negative, weak, moderate, and strong staining. The representative images of AnxA2 staining in tissues of normal, ER^+^ and/or PR^+^, HER2^+^, and TNBC patients are shown in Figure 1A. To verify the antibody specificity for AnxA2 and to exclude cross-reactivity with the other Annexin family proteins, we performed the immunoblotting using MDA-MB-231 (Control shRNA or AnxA2 shRNA knockdown) cell lysates. The AnxA2 antibody showed strong specificity for AnxA2 and no cross-reactivity were found with other Annexin family proteins (Figure S1). Based on molecular subtypes of breast cancer specimens, strong staining of AnxA2 was predominantly observed with only triple-negative subtypes of breast cancer specimens (54.54%, *n* = 18) compared with other breast cancer subtypes (*p* < 0.0001; Figure 1B). AnxA2 immunostaining was mainly localized at the membrane and with less extent in the cytoplasm of the tumor cells in TNBC specimens. In addition, specimens with the following characteristics showed high AnxA2 staining: negative ER and/or PR expression (*p* = 0.0062), negative HER2 (*p* = 0.0017) expression, and negative ER/PR/HER2 expression (*p* < 0.0001; Table 1).

### 2.2. Serum AnxA2 Levels in Breast Cancer Patients

**Figure 2:**
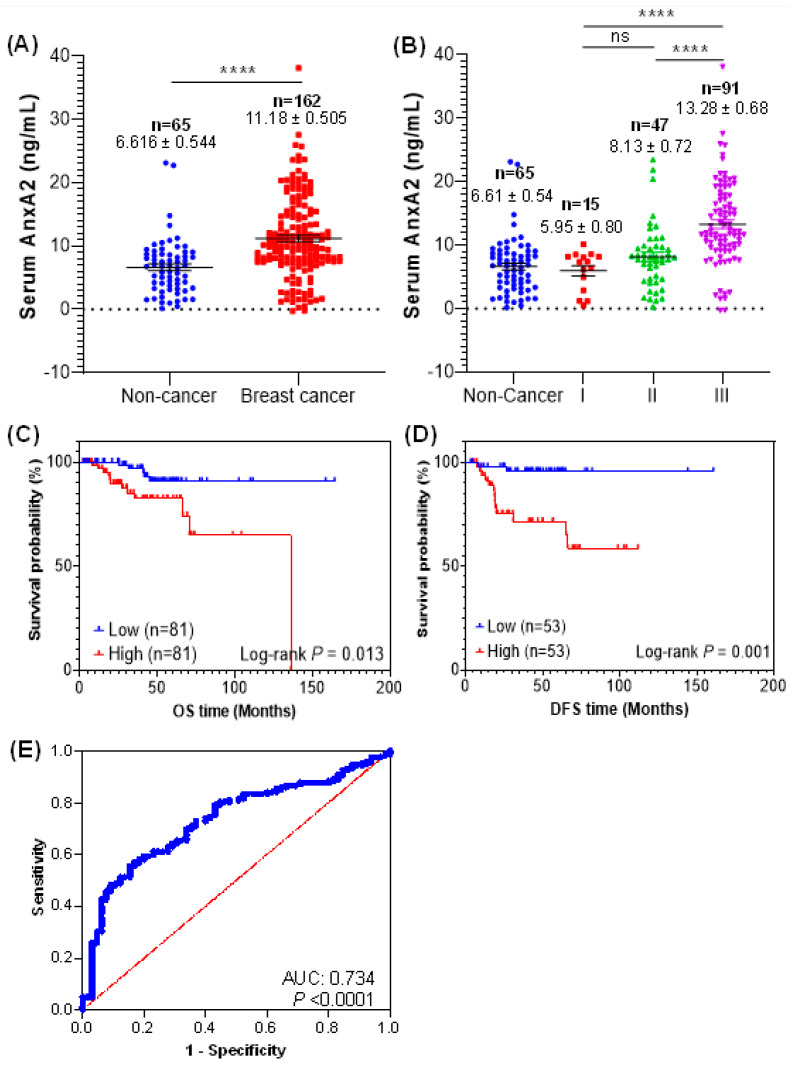
Serum AnxA2 levels in breast cancer patients. (**A**) Serum AnxA2 protein levels in breast cancer patients (*n* = 162) and normal healthy females (*n* = 65) were determined by ELISA. The data are presented as the mean ± SEM (****, *p* < 0.0001; two-tailed Students *t*-test). (**B**) Serum AnxA2 levels in different grades of breast cancer patients (Grade I, *n* = 15; grade II, *n* = 47; and grade III, *n* = 91). The data are presented as the mean ± SEM (^ns^, non-significant; ****, *p* < 0.0001; one-way ANOVA followed by Tukey multiple comparison test). Overall survival (**C**) and diseases-free survival (**D**) of breast cancer patients with high and low serum AnxA2 levels. The *p* value was calculated using the log-rank test. (**E**) ROC curve for breast cancer patients (*n* = 162) versus normal healthy females (*n* = 65).

The serum AnxA2 levels in breast cancer patients and normal healthy females were analyzed by ELISA. Our analysis showed that AnxA2 levels were significantly high in serum samples of breast cancer patients (*n* = 162; 11.18 ± 0.505 ng/mL, *p* < 0.0001) compared to normal healthy females (*n* = 65; 6.616 ± 0.544 ng/mL) (Figure 2A). In addition, the significant association between the serum AnxA2 levels and tumor grades were also observed in breast cancer patients (Figure 2B). The mean concentration of serum AnxA2 in normal healthy females was 6.616 ± 0.544 ng/mL (*n* = 65), whereas that in patients with grade I, II, and III breast tumor was 5.955 ± 0.800 ng/mL (*n* = 15, *p* = 0.9741), 8.135 ± 0.727 ng/mL (*n* = 47, *p* = 0.4624), and 13.28 ± 0.680 ng/mL (*n* = 91, *p* = 0.0001), respectively. The level of serum AnxA2 expression in grade III breast cancer patients were significantly higher than that of grade I and II patients (*p* < 0.0001), but no significant difference was observed between grade I and II breast tumor patients. Furthermore, we did not observe any significant difference in AnxA2 levels between healthy and grade I breast cancer patients. Together, these findings suggest that high levels of AnxA2 detected in serum samples of breast cancer patients is significantly associated with high tumor grades. Unlike tumor grade, no significant correlation between serum AnxA2 levels and tumor size, menopausal status, or lymph node metastasis was observed (Table 2).

### 2.3. Prognostic and Diagnostic Significance of Serum AnxA2 in Breast Cancer Patients

A Kaplan–Meier curves were used to analyze the prognostic role of serum AnxA2 levels in breast cancer patients. The median expression value of serum AnxA2 was categorized as a threshold to divide the breast cancer patients (*n* = 162) into two groups: high serum AnxA2 group (>10.11 ng/mL; *n* = 81) and low AnxA2 (<10.11 ng/mL; *n* = 81) group. As shown in Figure 2C, high serum AnxA2 group patients had significantly poor OS (Hazard ratio: 3.461; 95% confidence interval (CI) = 1.214–9.866; log-rank test *p* = 0.013) than those with low serum AnxA2 group. Similarly, the median expression value of serum AnxA2 was used for DFS evaluation in breast cancer patients (*n* = 106) and divide into high AnxA2 (>9.208 ng/mL; *n* =53) and low AnxA2 (<9.208 ng/mL; *n* = 53) groups. We saw that high serum AnxA2 group was associated with worse DFS (Hazard ratio: 8.160; 95% CI = 1.824–36.501; log-rank test *p* = 0.001) in breast cancer patients (Figure 2D). Taken together, our results suggest that high serum AnxA2 levels in breast cancer patients is associated with poor prognosis. To verify whether the serum AnxA2 could be used as a diagnostic marker for breast cancer, receiver operating characteristic (ROC) curves were used. The ROC curve based on serum AnxA2 values of breast cancer (*n* = 162) and normal healthy females (*n* = 65) showed that serum AnxA2 expression can be a significant parameter to discriminate between normal females and breast cancer patients with an area under the ROC curve (AUC) of 0.734 ± 0.035 (95% CI = 0.666–0.802; *p* < 0.0001) (Figure 2E). At the optimal cut-off value of 9.6 ng/mL, the sensitivity and specificity of detection was 84.6% and 55.6%, respectively.

### 2.4. Elevated Expression of Serum AnxA2 is Associated with TNBC Subtypes

**Figure 3:**
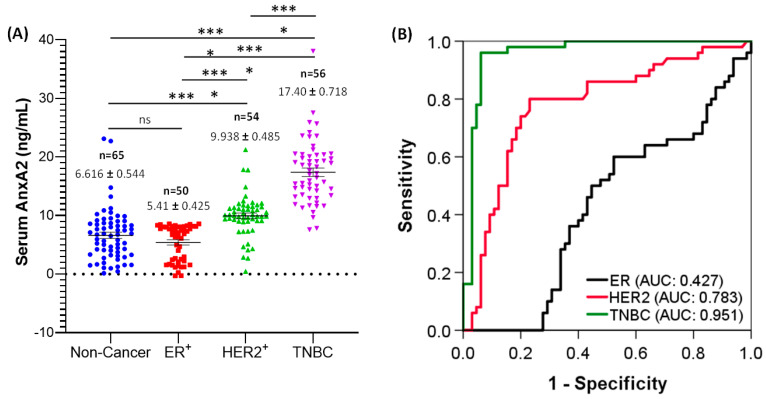
Serum AnxA2 levels in breast cancer subtypes. (**A**) Serum AnxA2 expression in normal healthy females (*n* = 65), ER^+^ (*n* = 50), HER2^+^ (*n* = 54), and TNBC (*n* = 56) breast cancer patients. The data are presented as the mean ± SEM (***, *p* < 0.001; ****, *p* < 0.0001; one-way ANOVA followed by Tukey multiple comparison test). (**B**) ROC curves for ER^+^ (*n* = 50), HER2^+^ (*n* = 54) and TNBC (*n* = 56) patients versus normal healthy females (*n* = 65).

The expression of serum AnxA2 levels in ER^+^, HER2^+^ and TNBC breast cancer patients were analyzed by ELISA assay. As shown in Figure 3A, the expression of serum AnxA2 was significantly elevated in TNBC (*n* = 56, 17.40 ± 0.718 ng/mL) in comparison to ER^+^ (*n* = 50, 5.410 ± 0.425 ng/mL, *p* < 0.0001), HER2^+^ (*n* = 54, 9.938 ± 0.485 ng/mL, *p* < 0.0001), and normal healthy females (*n* = 65, 6.616 ± 0.544 ng/mL, *p* < 0.0001). However, no difference was observed in serum AnxA2 levels between ER^+^ breast cancer patients and normal healthy females (Figure 3A). This analysis suggest that the expression of serum AnxA2 is predominantly associated with TNBC.

### 2.5. Serum AnxA2 Has Good Diagnostic Value for Triple-Negative Subtype of Breast Cancer

The level of serum AnxA2 in different breast cancer subtypes differed significantly. Compared with TNBC subtype, AnxA2 level in ER^+^ and HER2^+^ subtypes showed a significant decrease in patient serum (Figure 3A). Therefore, to elucidate the diagnostic value of serum AnxA2 in different breast cancer subtypes, ROC curve analysis based on serum AnxA2 levels of ER^+^, HER2^+^ and TNBC breast cancer patients compared to normal healthy patients was evaluated (Figure 3B). The ROC curve of serum AnxA2 showed that the AUC value of ER^+^ was 0.427 ± 0.054 (95% CI 0.322–0.533, *p* = 0.183), and a cut-off value of 6.0 ng/mL yielded 47.7% sensitivity and 60.0% specificity. For HER2^+^, the AUC value from ROC analysis based on the expression levels was 0.783 ± 0.044 (95% CI 0.697–0.869, *p* < 0.0001), and a cut-off value of 8.8 ng/mL yielded 76.9% sensitivity and 79.6% specificity. Similarly, a cut-off value of 11.3 ng/mL yielded 93.8% sensitivity and 92.9% specificity with an AUC of 0.951 ± 0.021 (95% CI 0.910–0.993, *p* < 0.0001) for predicting a triple-negative subtype (Figure 3B). These results indicate that serum AnxA2 level could be used as a potential diagnostic marker for TNBC patients.

### 2.6. AnxA2 Secretion Levels in Normal Mammary Epithelial Cell Lines and Breast Cancer Cell Lines with Different Subtypes

**Figure 4:**
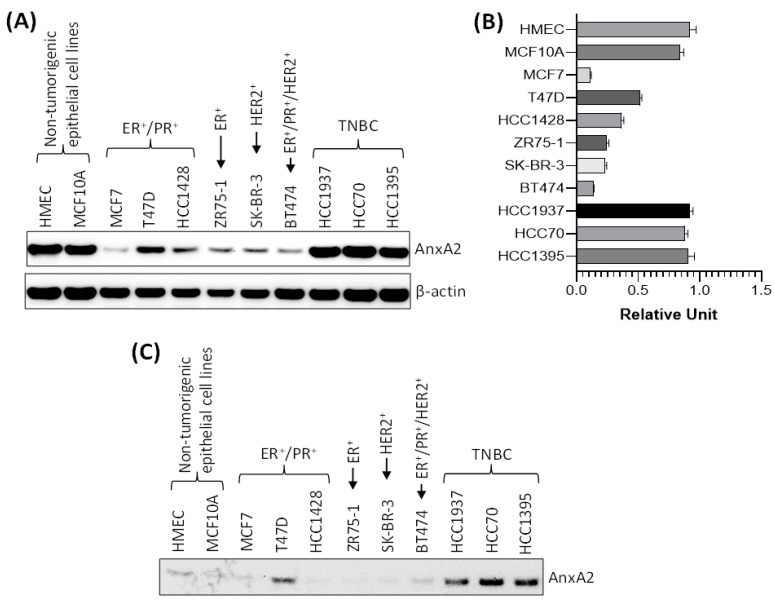
Expression and secretion of AnxA2 in breast cancer cell lines. (**A**) AnxA2 protein expression was analyzed by immunoblotting in breast cancer cell lines and non-tumorigenic mammary epithelial cell lines. The membrane was stripped and reprobed with anti-β-actin antibody for loading control. (**B**) Bar chart showing the densitometric analysis (using ImageJ software) of AnxA2 bands of the immunoblot of panel A. Intensity of AnxA2 bands was normalized by β-actin loading control. Each bar represents the mean ± SE of three independent experiments. (**C**) Non-tumorigenic or breast cancer cells (6 × 10^5^) were plated in 100mm Petri dish for overnight and then switched to their respective serum free medium. After 24 h, AnxA2 was immunoprecipitated from the medium using anti-AnxA2 antibody and expression of AnxA2 secretion was analyzed by immunoblot analysis. Uncropped Western Blots of (**A**,**C**) are available in Figure S2.

To further confirm that the high expression of AnxA2 found in tumor tissues and serum samples of TNBC patients are consistent with the breast cancer cell lines, we checked the expression and secretion of AnxA2 in non-tumorigenic mammary epithelial cell lines and in different subtypes of breast cancer cell lines. As shown in Figure 4A,B, the immunoblotting data clearly suggest that the expression of AnxA2 is high in TNBC cell lines (HCC1937, HCC70, and HCC1395 cells) compared to ER^+^ (ZR75-1 cells), ER^+^/PR^+^ (MCF7, T47D, and HCC1428 cells), HER2^+^ (SK-BR-3 cells), and ER^+^/PR^+^/HER2^+^ (BT474 cells) breast cancer cell lines. However, the expression of AnxA2 in TNBC cell lines are comparable to the non-tumorigenic mammary epithelial cell lines (HMEC, and MCF10A cells; Figure 4A,B). In another experiments, we compared the secretion of AnxA2 in breast cancer cell lines with normal mammary epithelial cells. The equal number of cells were seeded and grown in their respective medium for overnight and then switched to serum free medium. After 24 h, the AnxA2 was immunoprecipitated from culture medium using anti-AnxA2 antibody and expression of AnxA2 secretion was analyzed by immunoblot analysis. Our results suggest that the secretion of AnxA2 is high in TNBC cell lines compared to non-tumorigenic, ER^+^, PR^+^, and/or HER2^+^ breast cancer cell lines (Figure 4C). The results of the high secretion of AnxA2 from TNBC cell lines were further confirmed the conclusion derived from the serum samples of the breast cancer patients in this study, which suggest that high secretion of AnxA2 is specifically associated with the triple-negative subtypes of breast cancer patients.

### 2.7. Phosphorylation of AnxA2 at Tyr 23 is Associated with High Secretion of AnxA2 in TNBC

**Figure 5:**
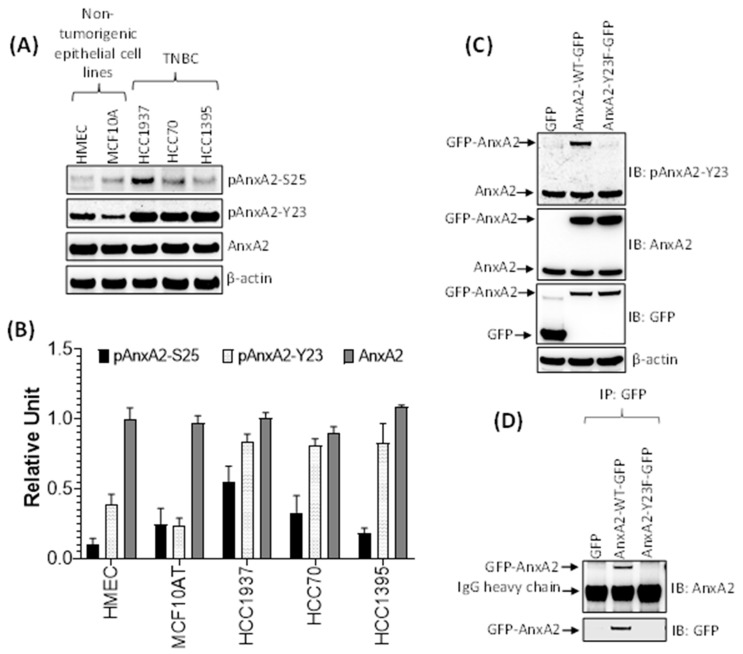
Phosphorylation of AnxA2 in breast cancer cells. (**A**) The expression of pAnxA2-S25, pAnxA2-Y23, and AnxA2 were analyzed in non-tumorigenic mammary epithelial cells and TNBC cells by immunoblot analysis. The membrane was stripped and reprobed with anti-β-actin antibody for loading control. (**B**) Bar chart showing the densitometric analysis of pAnxA2-S25, pAnxA2-Y23 and AnxA2 bands of the immunoblot of panel A. Intensity of each bands were normalized by loading control. Each bar represents the mean ± SE of three independent experiments. (**C**) The HCC1395 cells were transfected with a plasmid vector expressing GFP alone, AnxA2-WT-GFP, and AnxA2-Y23F-GFP. The expression of pAnxA2-Y23, AnxA2 and GFP were analyzed by immunoblot analysis. The membrane was stripped and reprobed with anti-β-actin antibody for loading control. (**D**) The HCC1395 transfected cells (6 × 10^5^) were plated in 100mm Petri dish for overnight and then switched to serum free medium. After 24 h, the AnxA2-GFP complex was immunoprecipitated from the medium using anti-GFP antibody (Catalog no. DSHB-GFP-12A6; DSHB) and expression of AnxA2 secretion was analyzed by anti-AnxA2 antibody using immunoblot analysis. Uncropped Western Blots of (**A**,**C**,**D**) are available in Figure S3.

**Figure 6:**
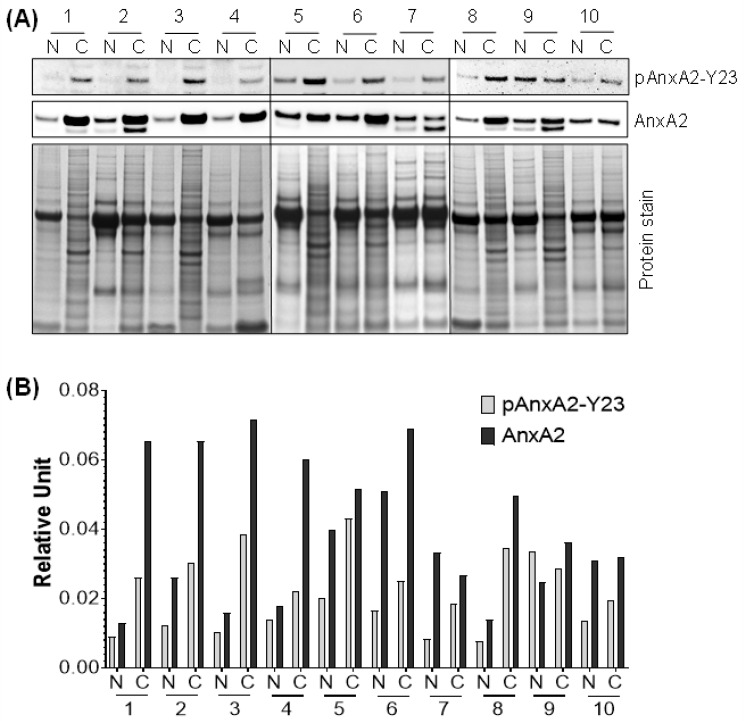
Expression of pAnxA2-Tyr23 and AnxA2 in tumor tissues of TNBC patients and matched adjacent non-tumorigenic breast tissues. (**A**) Immunoblot analysis of pAnxA2-Y23 and AnxA2 in tumor tissues of TNBC patients (C) and matching adjacent non-tumorous (N) breast tissues. Coomassie staining was used as a loading control. (**B**) Bar chart showing the densitometric analysis of pAnxA2-Y23 and AnxA2 bands of the immunoblot of panel A. Intensity of pAnxA2-Tyr23 and AnxA2 bands were normalized by total protein loading control. Uncropped Western Blots of (**A**)are available in Figure S4.

Our previous results showed that the secretion of AnxA2 is high in TNBC cell lines compared to non-tumorigenic mammary epithelial cell lines. However, the expression of endogenous AnxA2 is same in both cell types. Therefore, we next asked whether the phosphorylation status of AnxA2 an important event in the secretion process of AnxA2 from TNBC cell lines is. Because AnxA2 possesses two potential phosphorylation sites, Tyr23 and Ser25 at its N terminus, therefore, we checked the phosphorylation of these two sites in both non-tumorigenic mammary epithelial cell lines and TNBC cell lines using immunoblot analysis. The results presented in Figure 5A,B showed that the phosphorylation of AnxA2 at Tyr23 (pAnxA2-Y23) is very high in all TNBC cell lines (HCC1937, HCC70, and HCC1395 cells) compared to non-tumorigenic cell lines (HMEC, and MCF10A cells). The phosphorylation of AnxA2 at Ser25 (pAnxA2-S25) were only observed in HCC1937 TNBC cells whereas the other TNBC cell lines and non-tumorigenic cell lines were showing very minimal phosphorylation of AnxA2 at Ser25. However, the expression of AnxA2 is equal in both non-tumorigenic and TNBC cell lines (Figure 5A,B). To further check whether the phosphorylation of AnxA2 at Tyr23 is association with the secretion of AnxA2 from cells, we overexpressed wild-type AnxA2 tagged with GFP (AnxA2-WT-GFP), non-phosphomimetic mutant of AnxA2 tagged with GFP (AnxA2-Y23F-GFP), or GFP alone in HCC1395 cell line (Figure 5C). Immunoblot analysis showed that similar to endogenous AnxA2 phosphorylation at Tyr23 in HCC1395 cells, the overexpression of GFP tagged wild-type AnxA2 is also phosphorylated at Tyr23. In the case of the non-phosphomimetic Tyr23 mutant AnxA2-Y23F-GFP (Figure 5C), we could not detect any phosphorylation at Tyr23 in HCC1395 cells. To further check the role of Tyr23 phosphorylation in AnxA2 secretion, the conditioned media of HCC1395 cells transfected with AnxA2-GFP fusion construct were immunoprecipitated with anti-GFP antibody. On immunoblotting with anti-AnxA2 antibody, the secretion of GFP-AnxA2 were only observed in cells overexpressing AnxA2-WT-GFP while no GFP-AnxA2 expression were detected in cells overexpressing AnxA2-Y23F-GFP, indicating that phosphorylation of Tyr23 site is required for the secretion of AnxA2 (Figure 5D). To further determine whether high expression of AnxA2 detected in serum samples of TNBC patients is linked with the phosphorylation of AnxA2 at Tyr23 present in tumor tissues of TNBC patients, the expression of pAnxA2-Y23 were analyzed in tumor tissues of TNBC patients and matched adjacent non-tumorigenic breast tissues by immunoblot analysis. Compared with the non-tumorous corresponding tissues, the phosphorylation of AnxA2 at Tyr23 and total AnxA2 were observed in 90.0% (9/10) and 80.0% (8/10) of TNBC tumor tissue samples, respectively (Figure 6A,B). Together, these results suggest that high expression of AnxA2 found in serum samples of TNBC patients is associated with the high expression and phosphorylation of AnxA2 at Tyr23 in tumor tissues of TNBC patients.

## 3. Discussion

The expression and function of AnxA2 has been characterized in various malignancies including breast [33,34,35]. The overexpression of AnxA2 has been shown to play an important role in tumorigenic process such as proliferation, migration, invasion, adhesion, and angiogenesis [24,28,29,36,37,38,39,40,41]. In fact, AnxA2 expression has been identified as a potential biomarker for metastatic recurrence of breast cancer [32]. The results of the present study showed that AnxA2 is significantly overexpressed in tumor tissues of TNBC patients compared with ER and/or PR, HER2 patients and normal breast tissues. Notably, we found similar results with the serum samples of breast cancer patients, which illustrated as high circulatory level of AnxA2 in serum samples of TNBC patients compared to ER^+^, HER2^+^, and normal healthy females. The analysis of AnxA2 secretion and phosphorylation in a panel of breast cancer cell lines further suggest that the high secretion of AnxA2 from TNBC cells is primarily linked with the Tyr23 phosphorylation of AnxA2. Our studies with Tyr23 phosphorylation mutants of AnxA2 in HCC1395 cells clearly demonstrated that the phosphorylation of AnxA2 at Tyr23 possessed the high ability to secrete from cells, whereas the non-phosphomimetic mutant (Y23F) of AnxA2 failed to do so. These observations, in addition to previous findings [23], further suggest that AnxA2 phosphorylation at Tyr23 is an important and critical step for the secretion of AnxA2 from TNBC cells.

Despite the fact that AnxA2 does not have signals for its secretion [42], it was identified as a secretory biomarker for gastric cancer [43], oral squamous cell carcinoma [44], endometrial cancer [45] and hepatocellular carcinoma [46,47,48]. Although overexpression of serum AnxA2 was also observed in breast cancer [32], the possible clinical and diagnostic significance of serum AnxA2 in breast cancer patients has not been reported at present. The results of the present study further confirm that serum AnxA2 concentrations were elevated in breast cancer patients compared to normal healthy females. We observed that serum AnxA2 concentration increases with the progression of the breast tumor grade. In addition, Kaplan–Meier curves revealed that high expression of serum AnxA2 was significantly associated with poor overall survival and poor diseases-free survival of the breast cancer patients. Therefore, this suggest that circulating serum AnxA2 levels can be used as an independent prognostic factor for breast cancer patients. Furthermore, the results of the present study showed that circulating serum AnxA2 levels had the potential to distinguish breast cancer patients from normal healthy females, which suggest the potential value of serum AnxA2 as a diagnostic biomarker for breast cancer [32]. Consistent with studies reported previously in other cancers [43,44,45,46,47,48], the results of the present study further confirmed that the circulating concentrations of AnxA2 in serum could be used as a diagnostic and prognostic serological biomarker for breast cancer patients.

It was previously reported that the expression of AnxA2 increases with the aggressiveness of breast cancer [28,32]. The results of the present study in addition to our previous findings showed that AnxA2 is abundantly expressed in triple-negative subtypes of breast cancer at mRNA and protein levels [28,29]. The results of the present study demonstrated that the expression of serum AnxA2 is significantly elevated in TNBC compared to other subtypes of breast cancer patients. However, there is no information reported on the prognostic value of serum AnxA2 in TNBC patients. In the present study, we observed that the prognostic value of serum AnxA2 in comparison to normal healthy females is very high in TNBC (AUC = 0.951, *p* < 0.0001) compared to ER^+^ (AUC = 0.427, *p* = 0.1830) and HER2^+^ (AUC = 0.783, *p* < 0.0001) breast cancer patients. In addition, AnxA2 had a better sensitivity and specificity for TNBC at the optimal cut-off value compared to ER^+^ and HER^+^ patients. At this moment, it is difficult to establish the correlation between serum AnxA2 levels and clinical outcomes due to low number of patients in TNBC cohort. Taken together, these data suggest a potential application of serum AnxA2 as a diagnostic biomarker for TNBC.

## 4. Materials and Methods

### 4.1. Cell Lines and Culture Conditions

Human breast cancer cell lines MCF-7, T47D, HCC1428, ZR75-1, SK-BR-3, BT474, HCC1937, HCC-70, and HCC1395 and non-tumorigenic mammary epithelial cell lines HMEC, and MCF10A were purchased from the American Type Culture Collection (ATCC; Manassas, VA). MCF10A cells were cultured in DMEM/F12 medium supplemented with 5% horse serum, hydrocortisone (0.5 mg/mL), cholera toxin (100 ng/mL), insulin (10 μg/mL) and epidermal growth factor (EGF; 20 ng/mL). The primary human mammary epithelial cells (HMEC) were cultured in mammary epithelial growth medium (MEGM Bullet Kit; Lonza Corporation, Walkersville, MD). The MCF7, T47D, HCC1428, ZR75-1, HCC1937, HCC70, and HCC1395 cells were cultured in RPMI 1640 supplemented with 10% fetal bovine serum. MCF7, and T47D cells were further supplemented with human insulin (0.01 mg/mL). SK-BR-3 cells were maintained in McCoy’s 5a medium supplemented with 10% fetal bovine serum, while BT474 cells were maintained in high glucose DMEM medium containing 20% fetal bovine serum. All cell lines were maintained in a humidified incubator containing 5% CO_2_ and 95% air at 37 °C.

### 4.2. Materials

The cell culture medium RPMI 1640, DMEM, McCoy’s 5a, DMEM/F12, EGF, horse serum and fetal bovine serum were purchased from GIBCO (Invitrogen, Carlsbad, CA, USA). Antibodies against pAnxA2-Y23 (Catalog no. sc-135752), and β-actin (Catalog no. sc-47778) were obtained from Santa Cruz Biotechnology, Inc. (Dallas, TX, USA), whereas pAnxA2-S25 (Catalog no. PA5-37474) was from Thermo Fisher Scientific (Life Technologies Corporation, Grand Island, NY, USA). AnxA2 (clone 5; Catalog no. 610069) antibody was purchased from BD Pharmingen (BD Biosciences, San Jose, CA, USA). GFP antibodies for immunoblotting were purchased from Cell Signaling (Catalog no. 2955; Cell Signaling Technology, Inc., Danvers, MA, USA) and for immunoprecipitation experiments were purchased from Developmental Studies Hybridoma Bank, Iowa City, IA (Catalog no. DSHB-GFP-12A6). Protein A/G-agarose was acquired from Santa Cruz Biotechnology (Santa Cruz, CA, USA), and immunoblot stripping buffer from Pierce Co. (Rockford, IL, USA). All other reagents and biochemicals were purchased from Sigma-Aldrich.

### 4.3. Preparation of Cell Extracts and Immunoblot Analysis

Cells were lysed in radioimmunoprecipitation assay (RIPA) buffer (50 mM Tris–HCl, pH 7.5; 150 mM sodium chloride; 0.5% sodium deoxycholate; 1% Nonidet P-40; 0.1% sodium dodecyl sulfate) containing protease and phosphatase inhibitor cocktails (Millipore Sigma, MA, USA) at 4 °C. After sonication on ice, cell debris was removed by centrifugation at 12,000 × g for 10 min at 4 °C. Protein concentrations were determined by Pierce BCA protein assay kit (Thermo Scientific, IL, USA). Cell extracts were separated on 4–20% Bis-Tris Nu-PAGE gel (Invitrogen Corporation, CA, USA) and transferred onto nitrocellulose membrane. Membranes were blocked with 5% fat-free milk in Tris-buffered saline containing 0.05% Tween 20 (TBST) at room temperature for 60 min and incubated overnight at 4 °C with the appropriate primary antibody in 2% bovine serum albumin (BSA) in TBST. After three washings with TBST, the membranes were incubated with the appropriate secondary antibody (Southern Biotechnology, AL, USA) at room temperature for 2 h. After washing again with TBST, the membranes were developed using Immobilon Western Chemiluminescent HRP Substrate (Millipore Sigma, MA, USA), and the images were captured using alpha-imager Fluoretech HD2. The original Immunoblot data is shown in Figure S2.

### 4.4. Immunoprecipitation Studies

The cells (6 × 10^5^) were plated in 100mm Petri dish for overnight and then switched to serum free medium. After 24 h incubation, the medium was collected and incubated with anti-GFP (Catalog no. DSHB-GFP-12A6; DSHB) or anti-AnxA2 (Catalog no. 610069; BD Pharmingen) antibodies at 4 °C overnight. Protein A/G-PLUS agarose beads (60 μL) were then added to the medium and incubated for 2 h at 4 °C. The agarose beads were collected, washed three times with PBS, resuspended in 60 μL of 2× sample buffer, and boiled for 5 min to dissociate the immunocomplexes from the beads. The supernatant collected after centrifugation was subjected to immunoblot analysis.

### 4.5. Plasmids and Constructs

For the construction of a plasmid expressing full-length AnxA2, the cDNA of AnxA2 (NM_001002857) was cloned into the pLenti-C-mGFP-P2A-Puro vector (OriGene Technologies, Inc., Rockville, MD, USA). The N-terminal non-phosphomimetic mutant at Tyr-23 (AnxA2Y23F-GFP) was generated by site-directed mutagenesis. In this report, the plasmids are referred to as GFP, AnxA2WT-GFP, and AnxA2Y23F-GFP.

### 4.6. Tumor Tissues and Serum Samples Collection

Deidentified paraffin embedded breast cancer tissue microarray were procured from US Biomax, Inc. (*n* = 76) for IHC analysis, while frozen TNBC tumors and paired adjacent non-tumor breast tissue samples (*n* = 10) were obtained from Fox Chase Cancer Center Biosample Repository, Philadelphia, PA, USA. In addition, the archived serum samples of breast cancer patients (*n* = 162) and normal healthy females (*n* = 65) were collected from the Simmons Comprehensive Cancer Center, the University of Texas Southwestern Medical Center, Dallas, TX, USA. The samples were stored in freezer and were thawed on ice prior to use. All the archived and deidentified tumor tissues and serum samples were acquired under the Institutional Review Board (IRB) protocols approved by the University of North Texas Health Science Center, Fort Worth, TX, USA (IRB approval number: 2007-110, and 2013-016). The deidentified clinicopathological reports of serum samples, including tumor grade, receptor status (ER^+^, PR^+^, and HER2^+^), and survival of the patients were collected from the medical records of the breast cancer patients.

### 4.7. Immunohistochemical Staining

Annexin A2 expression was examined in ER^+^ and/or PR^+^ (*n* = 10), HER2^+^ (*n* = 24), TNBC (*n* = 33), and adjacent non-tumor breast tissue (*n* = 9) samples by immunohistochemistry (IHC) staining. Paraffin-embedded tissue sections (5 μm) were deparaffinized in xylene, rehydrated with a gradient of ethanol and washed in distilled water. Once deparaffinization was complete, endogenous peroxidase activity of the sections was quenched by incubation in BLOXALL^®^ Blocking Solution (Vector Laboratories, Burlingame, CA, USA) for 10 min at room temperature. After rinsing with TBS buffer, antigen retrieval step was performed using citrate buffer at 60 °C for 12 h followed by tissue permeabilization with 0.025% Triton X-100. In order to reduce the non-specific background staining, tissues were blocked by sequential incubation with avidin and biotin for 15 min each (Avidin/Biotin Blocking System; Vector Laboratories, Burlingame, CA, USA), followed by 30 min incubation with 2.5% normal horse serum (Vector Laboratories, Burlingame, CA, USA). Tissues were then incubated with anti-AnxA2 antibody (1:100 dilutions; Catalog no. 610069; BD Biosciences, San Jose, CA, USA) diluted in buffer containing 1.5% blocking serum. After washing, slides were incubated with a biotinylated Anti mouse secondary antibody for 30 min, followed by an enzyme-streptavidin-horse radish peroxidase conjugate for 20 min (R.T.U. VECTASTAIN Elite ABC Reagent; Vector Laboratories, Burlingame, CA, USA). Immunostaining visualization was achieved by immersion of slides in diaminobenzidine (DAB) (Vector Laboratories, Burlingame, CA, USA) for 5 min, followed by counterstaining with hematoxylin for 30 s. Slides were then dehydrated with increasing gradient of ethanol followed by incubation with xylene. The slides were covered with coverslips with Vectamount (Vector Laboratories, Burlingame, CA, USA) mounting medium. Sections incubated with PBS and without a primary antibody served as a negative control. The anatomic pathologist read the Hematoxylin (stains nuclei purple) and Eosin (stain acidophilic structures red or pink) (H&E) stained sections and AnxA2 stained sections to determine the intensity scores of AnxA2 for the normal breast tissue and tumor tissue sections. IHC scoring is performed as previously described [29,31]. Immunoreactivity was defined as score of 0 (no staining in all cells or very weak staining in less than 10% of the tumor cells), 1+ (weak partial staining in 10% to 30% of the tumor cells), 2+ (weak to moderate complete staining in more than 30% to 70% of the tumor cells) or 3+ (strong complete staining in more than 70% of the tumor cells).

### 4.8. Serum AnxA2 Quantification

AnxA2 concentrations in serum samples were measured by ELISA (Catalog no. DYC3928-2; R&D Systems, Inc., Minneapolis, MN) as performed previously [49]. Briefly, 96-well plate was coated with capture antibody at 4 °C for overnight. After washing, the plates were blocked with blocking buffer for 2 h at room temperature. The plates were incubated with serum diluted in buffer for 2 h at room temperature. The plates were washed and incubated with detection antibody for 2 h at room temperature. After washing, plates were incubated with Streptavidin-horseradish peroxidase for 20 min at room temperature, washed and further incubated with peroxidase substrate 3,3′,5,5′-Tetramethylbenzidine. The reaction was stopped using sulfuric acid (2N) and the optical density was measured at 450 nm with wavelength correction at 540 nm. Each sample was run in triplicates (*n* = 3).

### 4.9. Statistical Analysis

SPSS software 25 (SPSS Inc., IL) and GraphPad Prism 8 (GraphPad Software, CA, USA) were used for the statistical analysis. Scatter plots were used to plot the serum AnxA2 concentrations and results were presented as mean ± SEM. Comparison of means between two groups was calculated using Student’s *t* tests, while the comparison for more than two groups was conducted using one-way ANOVA. Data violating necessary assumptions were analyzed using non-parametric test. Categorical variables were compared using the χ² test. The overall survival (OS) and diseases-free survival (DFS) were calculated using the Kaplan–Meier method with the log-rank test applied for comparison [50]. Cox regression was used to compute hazard ratios and corresponding confidence intervals. Receiver-operating characteristic (ROC) curve and area under the curve (AUC) were used to assess the feasibility of using serum AnxA2 concentrations as a diagnostic tool for detecting breast cancer. The Youden’s J statistic was used to determine the cutoff value for serum AnxA2 concentrations [51]. Statistical significance was two-tailed and considered significant if *p* value was at least ≤ 0.05. (*), *p* < 0.05; (**), *p* < 0.01; (***), *p* < 0.001; (****), *p* < 0.0001.

## 5. Conclusions

In conclusion, our results demonstrated the several applications of serum AnxA2 concentrations in breast cancer patients in a non-invasive procedure. This study is innovative as it established the high diagnostic role of serum AnxA2 in the most aggressive subtype of breast cancer patients, TNBC. Altogether, these studies suggest the potential role of circulating AnxA2 in the diagnosis and prognosis of breast cancer patients.

## Figures and Tables

**Table 1:** Immunohistochemistry (IHC) scoring of breast tissue sections.

Type	Cases (*n*)	AnxA2 Expression (%)				χ^2^ and *p*-Value
		No Staining (Score = 0)	Weak Staining (Score = 1)	Moderate Staining (Score = 2)	Strong Staining (Score = 3)	
Normal/Malignant:						
Normal	9	5 (55.6)	3 (33.3)	1 (11.1)	0 (0)	χ^2^ = 12.55
Malignant	67	8 (11.9)	19 (28.4)	22 (32.8)	18 (26.9)	*p* = 0.0057
ER/PR:						
Positive	10	3 (30.0)	6 (60.0)	1 (10.0)	0 (0)	χ^2^ = 12.39
Negative	57	5 (8.8)	13 (22.8)	21 (36.8)	18 (31.6)	*p* = 0.0062
HER2:						
Positive	24	5 (20.8)	10 (41.7)	9 (37.5)	0 (0)	χ^2^ = 15.11
Negative	43	3 (7.0)	9 (20.9)	13 (30.2)	18 (41.9)	*p* = 0.0017
ER/PR/HER2:						
Triple-positive	34	8 (23.5)	16 (47.1)	10 (29.4)	0 (0)	χ^2^ = 35.07
Triple-negative	33	0 (0)	3 (9.1)	12 (36.4)	18 (54.5)	*p* < 0.0001

**Table 2:** The association of serum AnxA2 levels with clinicopathological characteristics in breast cancer patients.

Pathological Factors	Cases (*n*)	Serum AnxA2 Levels (Mean ± SEM, ng/mL)	Unpaired *t*-Test	*p*-Value
Tumor Size (cm):				
≤2	84	10.94 ± 0.696	0.061	*p* = 0.9512
>2	65	11.00 ± 0.718		
Menopausal Status:				
Premenopausal	28	10.01 ± 1.071		
Perimenopausal	8	10.11 ± 2.504	0.039	*p* = 0.9687
Postmenopausal	115	11.53 ± 0.616	1.119	*p* = 0.2651
Lymph Node Metastasis:				
Positive	58	9.946 ± 0.667	1.552	*p* = 0.1229
Negative	89	11.54 ± 0.708		
ER:				
Negative	110	13.74 ± 0.563	9.426	*p* < 0.0001
Positive	50	5.410 ± 0.425		
HER2:				
Negative	106	11.74 ± 0.724	1.683	*p* = 0.0943
Positive	54	9.938 ± 0.485		
ER/HER2 vs. TNBC:				
Triple-negative	56	17.40 ± 0.718	12.84	*p* < 0.0001
ER + HER2 positive	104	7.761 ± 0.392		

## Data Availability

The data presented in this study are available in the article and supplementary material.

## Supplementary Materials

Supplementary materials can be found at https://www.mdpi.com/2072-6694/13/1/2/s1. Figure S1: Validated of AnxA2 antibody by immunoblot analysis using wild-type and AnxA2 knock-down cell lysates. Figure S2: Uncropped Western Blots of Figure 4 (**A**) and (**C**), Figure S3: Uncropped Western Blots of Figure 5 (**A**), (**C**) and (**D**), Figure S4: Uncropped Western Blots of Figure 6 (**A**).
