# Supplementary material for: Clinical Significance of Annexin A2 Expression in Breast Cancer Patients
Source: Cancers (Basel). 2020 Dec 22;13(1):2. doi: 10.3390/cancers13010002 (PMC7792619; doi:10.3390/cancers13010002)
Supplement: Supplementary file 1 [file cancers-13-00002-s001.pdf]

Supplementary Material: Clinical Significance of Annexin A2 Expression in Breast Cancer Patients

Lee D. Gibbs, Kelsey Mansheim, Sayantan Maji, Rajesh Nandy, Cheryl M. Lewis, Jamboor K. Vishwanatha and Pankaj Chaudhary

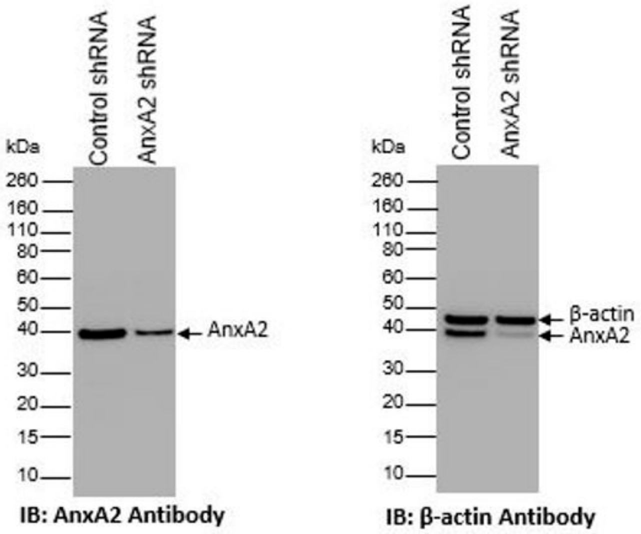

Figure S1. MDA-MB-231 cells were transfected with ANXA2 shRNA or control shRNA (Horizon Discovery, Cambridge, UK). After transfection, the expression of AnxA2 was determined by immunoblot analysis.

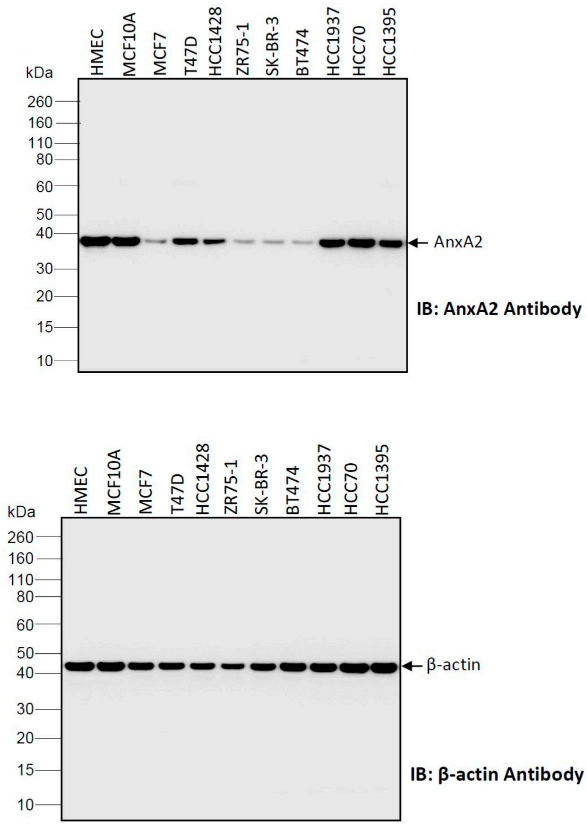

a

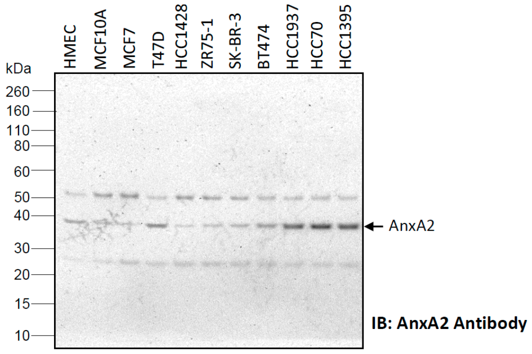

b

Figure S2. Uncropped Western Blots of Figure 4 (A) and (C). (**a**) imunoblot of Subfigure (A) and (**b**) imunoblot of subfigure (C).

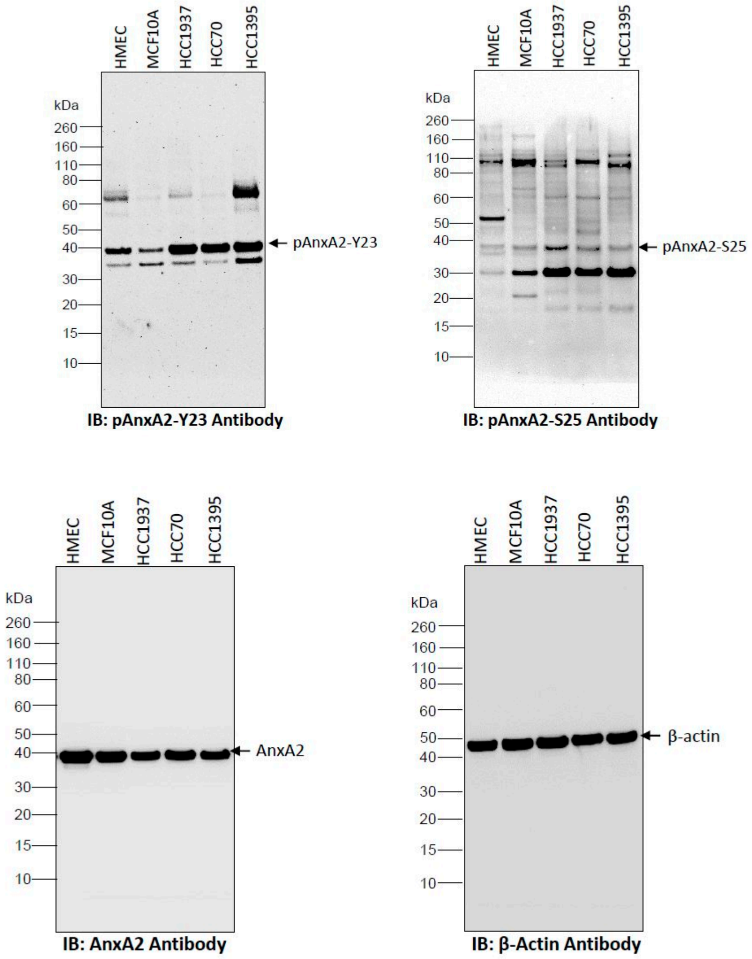

a

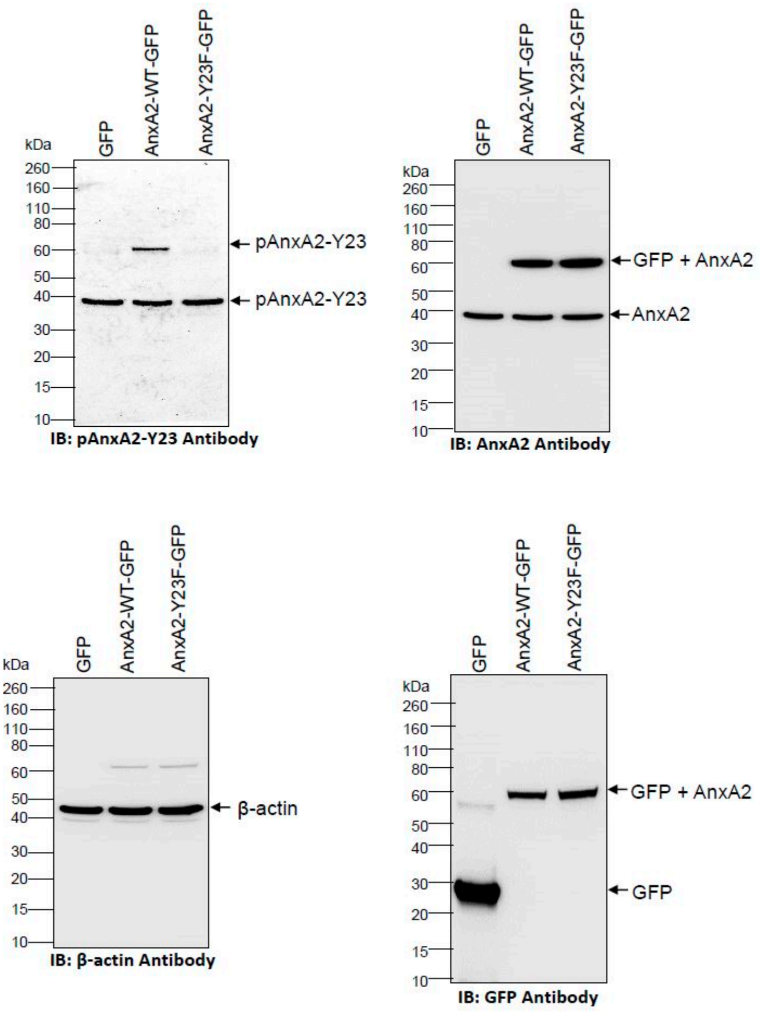

b

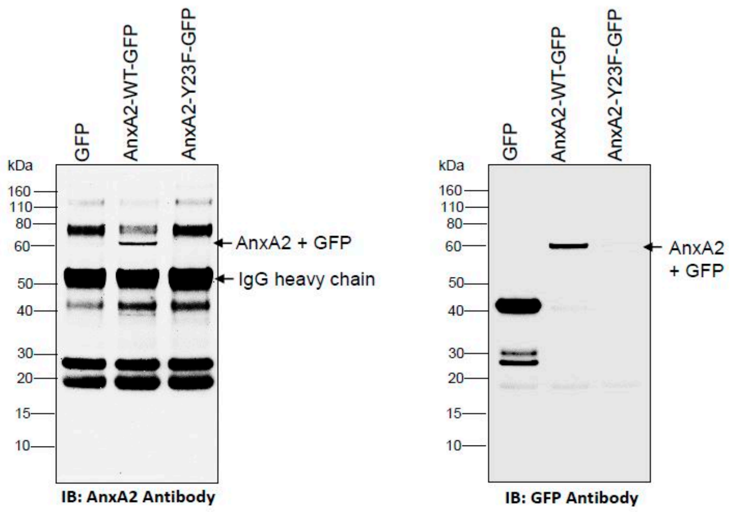

c

Figure S3. Uncropped Western Blots of Figure 5 (A), (C) and (D). (**a**) imunoblot of Subfigure (A). (**b**) imunoblot of Subfigure (C) and (**c**) imunoblot of Subfigure (D).

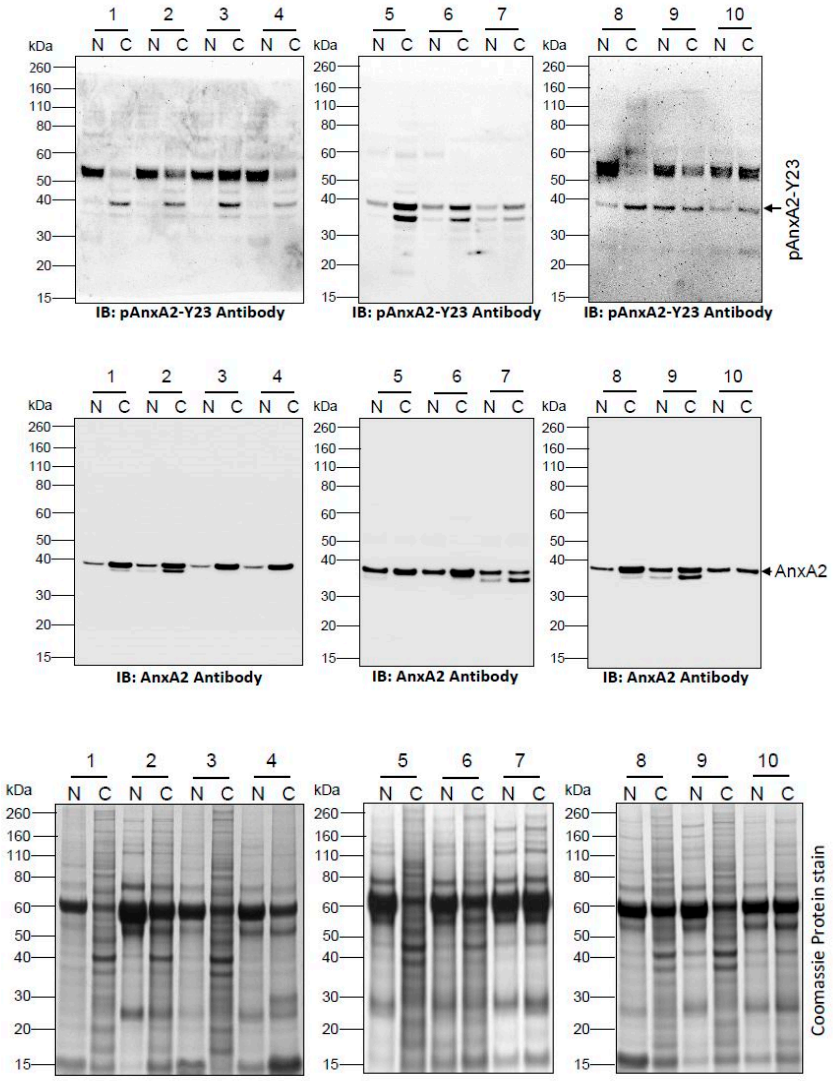

Figure S4. Uncropped Western Blots of Figure 6 (A).

Publisher's Note: MDPI stays neutral with regard to jurisdictional claims in published maps and institutional affiliations.

© 2020 by the authors. Licensee MDPI, Basel, Switzerland. This article is an open access article distributed under the terms and conditions of the Creative Commons Attribution (CC BY) license (http://creativecommons.org/licenses/by/4.0/).
